# 

**Published:** 1897-05

**Authors:** 


### CONTENTS.

**ORIGINAL COMMUNICATIONS.** PAGE

Crown- and Bridge-Work. By FRED. A. PEESO, D.D.S. . . . . . . . . . . . 285
Methods of Controlling the Electric Current in Cataphoresis. By WILLIAM ST. GEORGE ELLIOTT, JR. . . . . . . . . . . . . . . . . . . . . . . . . . . 297
Removable Porcelain Crown- and Bridge-Work. By ADAM FLICKINGER, D.D.S., St. Louis, Mo. . . . . . . . . . . . . . . . . . . . . . . . . . . . 306
"Not Failure, but Low Aim is Crime." By J. G. W. WERNER, D.M.D., Boston, Mass. . . . . . . . . . . . . . . . . . . . . . . . . . . . 310
Consolidation of the Two Associations. By DR. B. H. CATCHING, Atlanta, Ga. . 314

**ABSTRACTS AND TRANSLATIONS.**

The Discoloration of Copper Amalgam. By KENNETH W. GOADBY . . . . . . 315

**REPORTS OF SOCIETY MEETINGS.**

American Academy of Dental Science . . . . . . . . . . . . . . . . . 321
Academy of Stomatology . . . . . . . . . . . . . . . . . . . . . . . 332

**EDITORIAL.**

Then and Now . . . . . . . . . . . . . . . . . . . . . . . . . . . . 338
Dr. Bonwill's Visit to Europe . . . . . . . . . . . . . . . . . . . . 343
John C. Storey, M.D., D.D.S. . . . . . . . . . . . . . . . . . . . . 343
Edward Drinker Cope . . . . . . . . . . . . . . . . . . . . . . . . . 344
Professor Frank Abbott . . . . . . . . . . . . . . . . . . . . . . . 345

**BIBLIOGRAPHY** . . . . . . . . . . . . . . . . . . . . . . . . . . 345

**OBITUARY.**

John C. Storey, M.D., D.D.S. . . . . . . . . . . . . . . . . . . . . 349
Professor Edward Drinker Cope . . . . . . . . . . . . . . . . . . . . 350
Dr. Henry F. Blakeney . . . . . . . . . . . . . . . . . . . . . . . . 351

**NOTES AND COMMENTS** . . . . . . . . . . . . . . . . . . . . . . . 351

**CURRENT NEWS.**

Meeting of the American Medical Association . . . . . . . . . . . . . 353
Resolutions of American Academy of Dental Science . . . . . . . . . . 354
Board of Registration in Dentistry . . . . . . . . . . . . . . . . . . 355
Michigan Dental Association . . . . . . . . . . . . . . . . . . . . . 355
Recent Patents . . . . . . . . . . . . . . . . . . . . . . . . . . . . 356

